# Biosorption of Mercury (II) from Aqueous Solutions onto Fungal Biomass

**DOI:** 10.1155/2012/156190

**Published:** 2012-09-18

**Authors:** Víctor M. Martínez-Juárez, Juan F. Cárdenas-González, María Eugenia Torre-Bouscoulet, Ismael Acosta-Rodríguez

**Affiliations:** ^1^Área Académica de Medicina Veterinaria y Zootecnia, Instituto de Ciencias Agropecuarias, Universidad Autónoma del Estado de Hidalgo, Zona Universitaria, Rancho Universitario Km 1. C.P. 43600, Tulancingo de Bravo Hidalgo, Mexico; ^2^Laboratorio de Micología Experimental, Centro de Investigación y de Estudios de Posgrado, Facultad de Ciencias Químicas, Universidad Autónoma de San Luis Potosí, Avenida Dr. Manuel Nava No. 6, Zona Universitaria, 78320 San Luis Potosí, SLP, Mexico

## Abstract

The biosorption of mercury (II) on 14 fungal biomasses, *Aspergillus flavus* I–V, *Aspergillus fumigatus* I-II, *Helminthosporium* sp., *Cladosporium* sp., *Mucor rouxii* mutant, *M. rouxii* IM-80, *Mucor* sp 1 and 2, and *Candida albicans*, was studied in this work. It was found that the biomasses of the fungus *M. rouxii* IM-80, *M. rouxii* mutant, *Mucor* sp1, and *Mucor* sp 2 were very efficient removing the metal in solution, using dithizone, reaching the next percentage of removals: 95.3%, 88.7%, 80.4%, and 78.3%, respectively. The highest adsorption was obtained at pH 5.5, at 30°C after 24 hours of incubation, with 1 g/100 mL of fungal biomass.

## 1. Introduction

Heavy metal ion pollution has become wide spread throughout the world as a result of industrialization, which significantly threats the ecosystem, especially the people's health due to their severe toxicity. In order to minimize the impacts of metals contaminated, wastewaters need to be treated before discharge to water bodies. Environmental mercury levels have increased considerably in recent years. The direct anthropogenic sources of mercury in water bodies are related to numerous industrial applications (e.g., chloroalkali productions, pharmaceutical and cosmetic preparations, electrical instruments, and pulp and paper industries) and many products of common use (e.g., thermometers, batteries, and medical drugs) [1].

Mercury is one of the priority pollutants listed by the USEPA as it can easily pass the blood-brain barrier and affect the fetal brain [2]. High concentrations of Hg (II) cause impairment of pulmonary function and kidney, chest pain, and dyspnea [3–6]. The illness, which came to be known as Minamata disease, was caused by mercury poisoning gas as a result of eating contaminated fish. Mercury has very high tendency for binding to proteins and it mainly affects the renal and nervous systems [7]. Mercury removal from wastewaters needs to achieve very low levels for all these reasons. Metal sorption by different types of biomaterials such as inactive dried biomass of algae, bacteria, and fungi can serve for removing metals from solution because of their unique chemical composition [8–10] investigated the metal binding capacity of the thermophilic bacteria *Geobacillus thermodenitrificans*. According to this study, bacterial biomass reduced the concentration of Fe^3+^ (91.31%), Cr^3+^ (80.80%), Co^2+^ (79.71%), Cu^2+^ (57.14%), Zn^2+^ (55.14%), Cd^2+^ (49.02%), Ag^+^ (43.25%), and Pb^2+^ (36.86%) at different optimum pH at 720 min [11], also it was investigated the biosorption of cadmium (II) from aqueous solutions by industrial fungus *Rhizopus cohnii*, for this, some researchers reported the maximum uptake of cadmium by fungal biomass at 40.5 mg/g in optimal conditions, which was higher than many other adsorbents, including activated carbon. Some other researches also indicated that biosorption is a very effective method to remove metals from the water and wastewater [12–18]. The objective of this work was to study the removal of mercury (II) in solution by 14 species of fungi isolated from different areas of mine waste and resistant to various heavy metals. 

## 2. Experimental

### 2.1. Biosorbents

The biosorbents utilized were 14 fungal biomasses of *Aspergillus flavus* I–V, *Aspergillus fumigatus *I-II isolated from a mining waste in Zimapan, HGO, Mexico; *Helminthosporium *sp., *Cladosporium *sp.*, Mucor *sp. 1 and 2 resistant to zinc, lead, and copper isolated from the air collected near a zinc smelting plant in San Luis Potosi, S.L.P., Mexico; *Mucor rouxii *mutant resistant to copper and lead, obtained by mutagenesis with ethylmethanesulfonate; *Mucor rouxii* IM-80 (wild type), and *Candida albicans* isolated from a leather works, located in Leon, GTO Mexico.

### 2.2. Microorganism and Mercury (II) Solutions

The fungi were grown at 28°C in an agitated and aerated liquid mediUM containing thioglycolate broth, 8 g/L. After 4-5 days of incubation for *A. flavus* I–V, *A. fumigates *I-II, *Helminthosporium *sp., *Cladosporium *sp., *Mucor *sp 1-2, *M. rouxii* mutant, *M. rouxii*, IM-80, and *C. albicans*, the cells were centrifuged at 3000 rpm for 5 min, washed twice with trideionized water, and then dried at 80°C for 4 h in an oven. Finally, the fungal biomass was milled and stored in an amber bottle in the refrigerator until their use. 

For analysis were prepared a series of solutions of mercury of 100 mg/L, pH was adjusted with nitric acid, and the quantity of biomass added to each flask was of 1 g/100 mL for the mercury's solution. It taken samples at different times, the biomass is removed for centrifugation (3000 rpm/5 min), and the supernatant is analyzed to define the ion metal concentration.

### 2.3. Determination of Mercury (II)

The concentration of mercury ions in solution was determined spectrophotometrically at 492 nm using Dithizone (1,5-Diphenylthiocarbazone) as the complexing agent, by the formation of orange colored solution. The minimum detectable mercury concentration was 1.0 *μ*g/10 mL of dithizone solution [19].

## 3. Results and Discussion

### 3.1. The Effect of Incubation Time and pH

**Figure 1:**
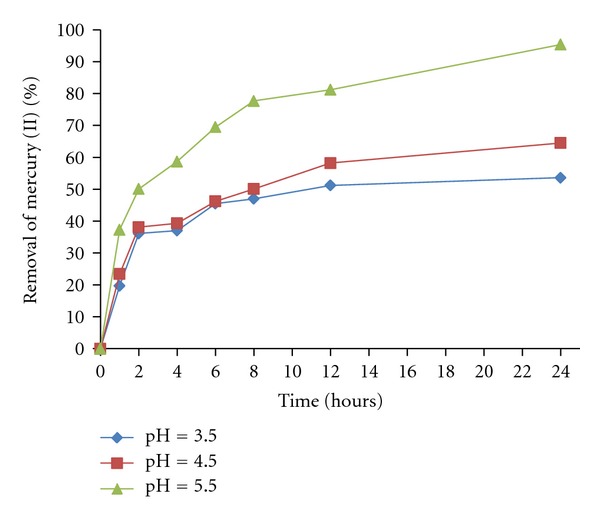
The effect of pH and incubation time on the biosorption of mercury (II). 100 mg/L Hg (II), 30°C, 100 rpm, 1 g of fungal biomass of *M. rouxii* IM-80.

Figure 1 shows the effect of contact time and pH on biosorption of Hg (II) ions (100 mg/L) to the dried *M. rouxii* IM-80 biomass, it was found that the highest removal occurred at 24 h of incubation and pH 5.5 (95.4%) (Figure 1), and these results resemble those reported by *Aspergillus versicolor* [20] and *Rhizopus oligosporus* [21]. Structural properties of the biosorbent including the cellular support and other several factors are known to affect the biosorption rate [22]. The pH is a critical parameter in biosorption because it influences the equilibrium by affecting the speciation of the metal ion(s) in solution, the concentration of competing hydrogen ions, and the chemistry of the active binding sites on the biomass. The fungal cell wall contains amino, carboxyl, and phosphate functional reactive groups. The carboxyl and phosphate groups carry negative charges that allow the fungal cell wall components to be potential detainer of metal ions [23]. The maximum biosorption of Hg (II) was observed at pH 5.5 (95.3%, Figure 1). At acidic pH (3.0), protonation of the cell wall components adversely affected the biosorption capacity of the fungal biomass, but its effect became minor with increasing pH in the medium. With an increase in pH, the negative charge density on the cell surface increases due to the deprotonation of the metal binding sites and thus increases biosorption [23]. Several researchers investigated the effect of pH on biosorption of mercury (II) by using different kinds of microbial biomasses. For example, *A. versicolor* [20], *R. oligosporus* [21], *Penicillium purpurogenum* [23], and the maximum biosorption were obtained in the pH range of 5.0 to 7.0.

### 3.2. Effect of Temperature

**Figure 2:**
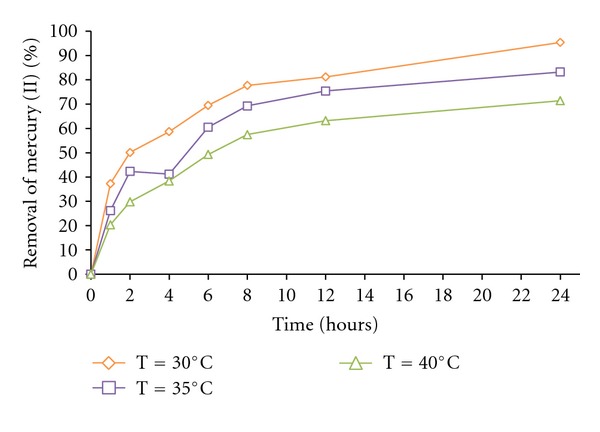
The effect of the temperature on mercury (II) removal. 100 mg/L Hg (II), 100 rpm. pH 5.5, 1 g of fungal biomass of *M. rouxii* IM-80.

Figure 2 shows the effect of varying temperatures (30°C, 35°C, and 40°C), the maximal adsorption capacity was found at 30 ± 1°C, (95.3%), and the adsorption capacity of dried *M. rouxii* IM-80 biomass decreased with temperatures higher than 30 ± 1°C (83.2% at 35°C, and 71.4% at 40°C). This is like to the report for *A. versicolor*, *R. oligosporus*, and* Bacillus subtilis *[20, 21, 24]. The temperature of the adsorption medium could be important for energy-dependent mechanisms in metal biosorption by microorganisms. Energy-independent mechanisms are less likely to be affected by temperature since the process responsible for biosorption is largely physicochemical in nature. The biosorption of Hg (II) by *M. rouxii* IM-80 fungus appears to be temperature-dependent over the temperature range tested (30–40°C).

### 3.3. Effect of Initial Mercury (II) Concentration

**Figure 3:**
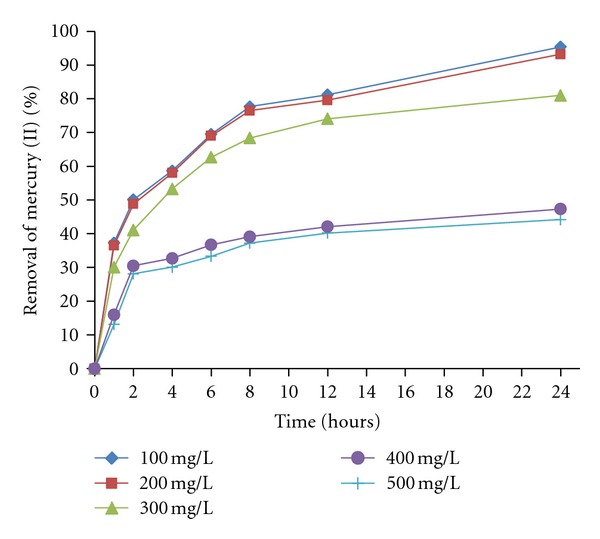
The effect of the concentration of mercury (II) in solution on the removal of Hg (II) ions. 100 rpm, 30°C, pH 5.5.1 g of fungal biomass of *M. rouxii* IM-80.

Biosorption capacities of the *M. rouxii* IM-80 biomass for the mercury (II) ions were studied as a function of the initial Hg (II) ions concentration between 100 and 500 mg/L in the biosorption medium (Figure 3). Although the percentage of adsorption decreased, when ions concentration increased. A similar type of trend was reported for the removal of Hg (II) from aqueous solution by sorption on *R. oligosporus* [21], *B. subtilis* [24], *Pleurotus sapidus* [25], biogenic silica modified with L-cysteine [26], and activated carbon prepared from agricultural byproduct/waste [6]. These results may be explained to be due to the increase in the number of ions competing for the available binding sites and also because of the lack of active sites on the biomass at higher concentrations [6].

### 3.4. Effect of Initial Biomass Concentration

**Figure 4:**
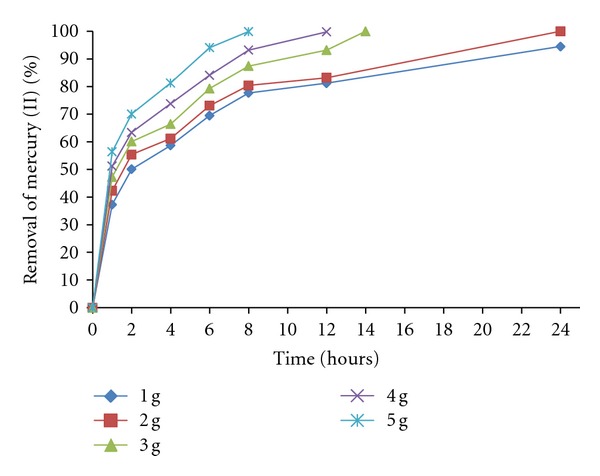
The effect of fungal biomass concentration on the removal of mercury (II). 100 mg/L, mercury (II), 100 rpm, 30°C, pH 5.5.

The influence of biomass on the removal capacity of mercury (II) was depicted in Figure 4. If we increase the amount of biomass also increases the removal of the metal in solution (100% of removal, with 5 g of fungal biomass, at 8 hours), with more biosorption sites of the same, because the amount of added biosorbent determines the number of binding sites available for metal biosorption [27]. Similar results have been reported for *Acetobacter xylinum* cellulose [27], *Mucor racemosus *biomass [28], and *Saccharomyces cerevisiae* [29].

#### 3.4.1. Biosorption of Mercury (II) By Different Fungal Biomasses

**Figure 5:**
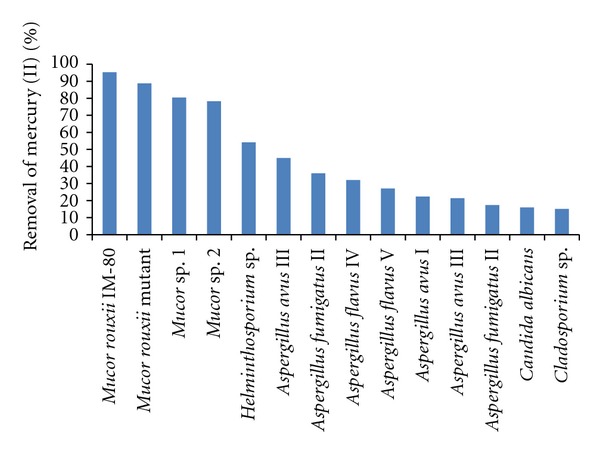
Biosorption of mercury (II) on different fungal biomasses. 100 mg/L Hg (II), 100 rpm, 30°C, pH 5.5, 1 g of fungal biomass.

In Figure 5, we show the biosorption of mercury (II) by the different biomasses analyzed. It was found that the biomass of the fungus *M. rouxii* IM-80, *M. rouxii *mutant, *Mucor *sp1, and *Mucor *sp2 were very efficient at removing the metal in solution (95.3%, 88.7%, 80.4%, and 78.3%, resp.). We do not know why the fungal biomasses of the mucorales were the most efficient at removing mercury (II) in solution. However, this difference may be because the polysaccharides of the cell wall could provide binding groups including amino, carboxyl groups and the nitrogen and oxygen of the peptide bonds could be accompanied by displacement of protons, dependent in part upon the extent of protonation as determined by the pH [21–23, 30]. 

Otherwise, in mercury detoxification process, work is still necessary to illustrate the distribution and diversity of the microbial communities under heavy metals stress in order to employ them for the bioremediation of these toxic pollutants, singly or in combination for greater efficiency [31]. Moreover, some mercury biosorbent fungi cannot only detoxify mercury but also remove other metals such as cadmium, chromium (VI), and lead [32].

## 4. Conclusion

In this study, mercury uptake by different fungal biomasses was investigated. The performance of the biosorbents was examined as a function of the operating conditions, in particular incubation time, pH and initial metal ion concentration, and fungal biomass. The experimental evidence shows a strong effect of the experimental conditions. Maximum biosorption capacity values showed that some biosorbents used are very effective in recovery or removal of mercury ion from aquatic systems. When the ease of production and economical parameters are concerned, it was observed that *M. rouxii* IM-80, *M. rouxii *mutant, *Mucor *sp. 1, and *Mucor *sp. 2 are a very promising biomaterial for removal or recovery of the metal ion studied.
